# Anticipatory Advance Care Planning Visits and COVID-19 Treatment Intensity Among Medicare Fee-for-Service Beneficiaries: A Retrospective Observational Study

**DOI:** 10.1007/s11606-025-09638-9

**Published:** 2025-12-12

**Authors:** Amber E. Barnato, Deanna L. Chyn, Vrushabh P. Ladage, Ellen Meara

**Affiliations:** 1 The Dartmouth Institute for Health Policy & Clinical Practice, Geisel School of Medicine at Dartmouth, Lebanon, NH USA; 2 Section of Palliative Care, Department of Medicine, Geisel School of Medicine and Dartmouth Health, One Medical Center Drive, Lebanon, NH 03756 USA; 3 Department of Health Policy and Management, Harvard T. Chan School of Public Health, 677 Huntington Ave, Boston, MA 02115 USA; 4 National Bureau of Economic Research, 1050 Massachusetts Ave, Cambridge, MA 02138 USA

**Keywords:** COVID-19 pandemic, advance care planning, serious illness, mechanical ventilation

## Abstract

**Background:**

In March 2020, professional organizations issued guidelines for anticipatory COVID-19 advance care planning (aACP) with high-risk older adults.

**Objective:**

To examine responses to these guidelines and associated COVID-19 treatment intensity.

**Design:**

Retrospective regression discontinuity design (RDD) using 2020 Medicare Parts A and B claims to assess aACP receipt, by beneficiary COVID-19 mortality risk. Adjusted logistic regression to assess predictors of aACP and the association between aACP and COVID-related mechanical ventilation.

**Patients:**

Medicare fee-for-service beneficiaries 66 and older enrolled on January 1, 2020.

**Main Measures:**

ACP visits assessed via current procedural terminology billing codes 99497 and 99498: classified as anticipatory if the place of service was ambulatory and outside an annual wellness visit. COVID-19 mortality risks based on age and/or co-morbidity and practice attribution were assessed pre-lockdown. Hospitalization within 2 weeks of COVID-19 diagnosis and associated mechanical ventilation (MV) were assessed post-lockdown.

**Key Results:**

In total, 24,935,234 beneficiaries received 470,046,404 encounters in 2020; 1,578,331 were for ACP; of these, 318,813 (20%) were classified as anticipatory. Lockdown abruptly decreased all encounters. The RDD estimate found aACP decreased by 54% (95% CI= −0.62, −0.46); the relative decline in aACP was smaller for highest COVID-19 mortality risk groups: multimorbidity (−42%; 95% CI= −0.48, −0.35), patients with advanced cancer (−44%; 95% CI= −0.52, −0.36), dementia (−35%; 95% CI= −0.43, −0.27), or ESRD (−38%; 95% CI= −0.55, −0.21). Care from an integrated health system was associated with less aACP. Among 1,314,986 beneficiaries diagnosed with COVID-19, aACP was associated with an increase in adjusted 14-day hospitalization (OR = 1.21; 95% CI=1.17–1.26) but a decrease in invasive mechanical ventilation if hospitalized (OR = 0.85; 95% CI=0.77–0.96).

**Conclusions:**

Providers followed recommendations to conduct anticipatory COVID-19 ACP with their high-risk patients, which was associated with reduced COVID-19 treatment intensity. This underscores the need for effective identification and dissemination of clinical risk factors in the next pandemic to guide anticipatory decision making.

**Supplementary Information:**

The online version contains supplementary material available at 10.1007/s11606-025-09638-9.

In March 2020, in response to reports from Italy and Switzerland regarding hospitalization and exceptionally high case fatality rates among older adults with the novel coronavirus disease 2019 (COVID-19), thought leaders called for primary care and specialty clinicians to proactively discuss preferences for life-sustaining treatment in the event of infection with their patients who were at highest risk for COVID-19 associated mortality, such as those at older ages with chronic, life-limiting illnesses.^1^ It is not known whether practicing clinicians followed these recommendations to conduct proactive, “anticipatory” COVID-19 ACP with their high-risk patients.

ACP is a process that allows individuals to discuss and potentially document their values, life goals, and treatment preferences to inform future medical care in the event they are unable to participate in medical decision making. The goal of ACP is to ensure goal-concordant treatment. Beginning in January 2016, the Centers for Medicare & Medicaid Services (CMS) introduced Current Procedural Terminology (CPT) reimbursement codes for ACP visits (99497 and 99498). The introduction of these codes signaled recognition of the importance of ACP in clinical practice and provided an opportunity to track trends in billed service use. In this study, we sought to measure the frequency of ACP billing among high-risk Medicare beneficiaries in the early phase of the pandemic, assess whether mortality risk influenced anticipatory ACP, and explore the association between anticipatory ACP and COVID-related invasive mechanical ventilation. Based upon anecdotal reports, we hypothesized that providers targeted high-risk patients for anticipatory COVID-19 ACP and that patients who received anticipatory ACP would be less likely to receive invasive mechanical ventilation if hospitalized with COVID-19. We hypothesized targeted anticipatory ACP would be more easily facilitated, and thus more common, in integrated health systems with broader provider networks and advanced health information technology.

## METHODS

### Design Overview

**Figure 1:**
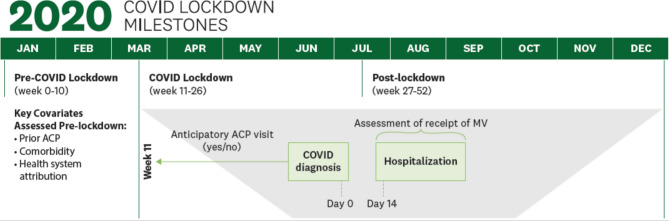
Study assessment time points. We characterized the pre-COVID lockdown period as January 1, 2020, to March 15, 2020 (week 11), and the lockdown period as March 15, 2020, to June 28, 2020 (week 26). March 15, 2020, corresponds to the first full week following the March 13^th^ declaration of a national emergency in the USA. June 28, 2020, corresponds to the last full week in June, by which most states had lifted stay-at-home orders. For the analysis of the association between anticipatory ACP and COVID-related mechanical ventilation, the anticipatory ACP had to occur before the hospitalization, the hospitalization had to start within 14 days of a COVID-19 diagnosis, and the mechanical ventilation could occur any time during the hospitalization.

We characterized the pre-COVID lockdown period as January 1, 2020, to March 15, 2020 (week 11), and the lockdown period as March 15, 2020, to June 28, 2020 (week 26). March 15, 2020, corresponds to the first full week following the March 13^th^ declaration of a national emergency in the USA. About half of US states had stay-at-home orders in place through May 2020 and only two states (New Mexico and California) had active stay-at-home orders in the final week of June 2020.^2^ June 28, 2020, corresponds to the last full week in June in this analysis of weekly health care utilization; and thus, it was chosen as the end of the lockdown period. We summarize exposure and outcome time periods in Figure 1.

We conducted a retrospective regression discontinuity design (RDD) using 2020 Medicare Parts A and B claims assessing beneficiary COVID-19 mortality risk (measured pre-lockdown) and anticipatory ACP after lockdown. This design leveraged the abrupt, exogenous change in health care services (in this case ACP) that occurred after week 11, as compared with weekly trends leading up to that time, following the March 13, 2020, declaration of COVID-19 as a National Emergency when widespread policy changes and regulatory guidance were issued. RDD can approximate the conditions of a randomized controlled trial (RCT) where “treatment” is assigned randomly, enabling researchers to estimate the causal effect. We used logistic regression to explore predictors of anticipatory ACP and hospitalization with invasive mechanical ventilation (MV) within 2 weeks of COVID-19 diagnosis after lockdown.

### Human Ethics and Consent to Participate

The study was reviewed and approved by the Dartmouth Committee for the Protection of Human Subjects. The requirement to obtain consent was waived under exemption 4 of the Common Rule (45 CFR 46).

### Patients

Patients included 100% of fee-for-service (FFS) Medicare beneficiaries aged 66 years and older continuously enrolled from January 1, 2020, to December 31, 2020, or until their date of death, residing in the 50 US states or Washington, DC.

We characterized patients’ sociodemographic characteristics, including race, ethnicity, area deprivation index (ADI), and rural residence using Medicare enrollment files. Race/ethnicity categories were based upon the RTI race code (American Indian/Alaskan Native, Asian Pacific Islander, Black or African American, Non-Hispanic White, Other, Unknown, Hispanic (any race)). We included race and ethnicity to capture socioeconomic/system predictors, such as the cumulative effects of racism on a person’s health, since race is a social, not a biological, construct. ADI is based on 17 census-tract level measures such as median household income, percent in poverty, percent with a high school degree, and median home value, drawn from the US Census Bureau.^3^ We operationalized this for our analysis by first assigning ZIP codes to quintiles of ADI (based on the continuous ADI value) and then rolling up the quintiles into three groups: “least disadvantaged” (quintile 1), “somewhat disadvantaged” (quintiles 2–4), and “most disadvantaged” (quintile 5); beneficiaries inherited the categorization of their ZIP of residence. We aggregated these measures to the ZIP code level, and we confirmed that the ADI index at the tract and ZIP code level has a correlation of 0.77 (*p*-value < 0.001). Rural residence was assigned based upon rural-urban commuting area (RUCA) codes assigned to each ZIP code (1 representing most urban, 10 most rural) based on measures of population density, urbanization, and daily commuting from the United States Department of Agriculture Economic Research Service.^4^ Beneficiaries were categorized as rural if they resided in a ZIP code having a RUCA value of 10; beneficiaries in all other ZIPs were considered non-rural. The rationale for this strict definition of rurality was to distinguish rural areas that were less likely to have access to reliable broadband and less likely to be located near large systems of health care providers.

### Main Measures

#### Advance Care Planning Visits

We identified 2020 Parts A and B Medicare fee-for-service visits for ACP using current procedural terminology (CPT) billing codes 99497 and 99498. We further characterized where ACP occurred by using place of service codes. If the place of service was the hospital, emergency department, or nursing home, we assumed that the conversation might be in response to active health deterioration and therefore related to “in the moment” proximate decision making, known as “goals of care” conversations or “care planning” (i.e., reactive instead of proactive). If the place of service was the non-acute, outpatient setting, we assumed that the ACP visit was, indeed, planning for hypothetical care decisions in the future. Because ACP is a standard part of the annual wellness visit (AWV), we excluded ACP completed during an AWV in our definition of “anticipatory” ACP, since we sought to capture ACP visits that represented targeting of high-risk patients for “extra” ACP. Thus, ACP visits were classified as anticipatory if the place of service was not the hospital, emergency department, or nursing home, and the visit was not an AWV. We hypothesized that if the anticipatory ACP occurred after lockdown, then they could plausibly have been COVID-19 anticipatory. In sensitivity analyses, we explored the influence of classifying ACP conversations in the nursing home or during AWV as potentially COVID-19 anticipatory*.*

#### COVID-19 Mortality Risk

The National Hospice and Palliative Care Organization (NHPCO) released an advance care planning (ACP) decision-making tool to guide patients to self-assess their risk of mortality in the event of illness and use that information to inform advance directive completion.^5^ Using NHPCO guidelines, we classified patients as “high-risk” if they were aged 80 and older with at least one serious chronic condition that could heighten COVID-19 mortality risk or if they were any age with multimorbidity (two or more serious chronic conditions). Additional “high-risk” populations of interest included patients with conditions frequently mentioned in ventilator allocation triage guidelines, including poor-prognosis cancers, end-stage renal disease (ESRD), and dementia.^6^

We identified chronic condition categories based upon their contribution to COVID-19 mortality risk per the Centers for Disease Control and Prevention case report form,^7^ including coronary artery disease, stroke, congestive heart failure, chronic obstructive pulmonary disease, diabetes, hematologic/thrombotic disease, HIV/AIDS, immune disease, liver disease, Parkinson’s/Huntington’s disease, paralysis, renal disease, severe mental illness, and peripheral vascular disease. In addition, we selected poor-prognosis cancers, ESRD, and dementia because these conditions were listed as categorical exclusions in multiple ventilator allocation triage guidelines.^6^ We defined poor prognosis cancers using International Statistical Classification of Diseases, Tenth Revision (ICD-10) codes mapped by Wasp based on Iezzoni et al.^8^ We identified dementia based on at least one ICD-10 dementia diagnosis from an encounter using inpatient, carrier, and outpatient Medicare fee-for-service claims.^9^ All other diagnoses were defined using hierarchical condition categories (HCCs). Given the lack of a full 1-year lookback period, we characterized a beneficiary as having the condition if they met the claims-based definition in the pre-lockdown period. Using 2019 data, we found that HCC capture using weeks 0 to 10 versus 0 to 52 was sensitive and specific for our conditions of interest. In addition to categorical variables for these select conditions, we calculated an HCC score for each beneficiary using all conditions based upon claims during the pre-lockdown period.

#### System Attribution

We used the IQVIA OneKey database to link providers on claims to health systems and practices using the National Provider Identifier and classified health systems as integrated (an organization that owns one or more hospitals and two or more medical practices) versus an independent medical practice or medical group using methods described previously.^10–12^ We used claims to attribute each patient to a health system on the basis of the plurality of primary care visits. We adapted our attribution method to restrict to the pre-lockdown weeks 0 to 10 in addition to the full-year attribution (weeks 11–52). Because full-year attribution involved using encounters that occurred after the outcome of interest was measured (anticipatory ACP during lockdown), we elected to use attribution based on weeks 0 to 10 in our primary models.

#### COVID-19 Diagnosis, Hospitalization, and Mechanical Ventilation

We identified beneficiaries with COVID-19 using ICD-10 code B9729 from January to March and U071 thereafter. We identified hospitalization within 2 weeks of a COVID-19 diagnosis from inpatient and skilled nursing facility claims occurring at short-term or critical access hospitals. We identified invasive mechanical ventilation during the hospitalization using ICD-10 codes 5A1935Z, 5A1945Z, and 5A1955Z.

### Analysis

#### Anticipatory ACP Visits

To explore whether COVID-19 mortality risk influenced anticipatory ACP, we used a fuzzy regression discontinuity design comparing anticipatory ACP visits prior to and after March 15, 2020 (week 11), until June 28, 2020 (week 26), among higher and lower mortality risk beneficiaries. We estimated linear regression models with a linear “weeks” trend for weeks 1 to 10, an indicator for weeks ≥11 (level change) and a linear “weeks” trend equal to 0 in weeks < 11 (trend change).

Separately, we used logistic regression to explore predictors of anticipatory ACP in the lockdown period among all beneficiaries: age, comorbidity, and receipt of any ACP in weeks 0 to 10. We also fit a supplemental model that added observable variables associated with socioeconomic and systemic (structural) factors that could be correlated with COVID-19 exposure risk, unmeasured comorbidity, and/or access to medical care, including race and ethnicity, Medicaid enrollment, a measure of area deprivation (area deprivation index (ADI)), and system attribution.

#### COVID-19 Hospitalization and Mechanical Ventilation

To explore whether anticipatory ACP influenced receipt of life-sustaining treatment in the event of COVID-19, we used logistic regression to explore the association between anticipatory ACP and hospitalization from the start of lockdown (week 11) through the remainder of 2020 (week 52), and, conditional upon hospitalization within 14 days of a COVID-19 diagnosis, whether the patient received invasive mechanical ventilation during the hospitalization. We adjusted regressions for age, comorbidity, and pre-lockdown ACP. The anticipatory ACP had to occur before the hospitalization, the hospitalization had to start within 14 days of a COVID-19 diagnosis, and the mechanical ventilation could occur any time during the hospitalization (see Fig. 1). As above, we also fit a supplemental model that added observable variables associated with socioeconomic and systemic (structural) factors that could be correlated with COVID-19 exposure risk, unmeasured comorbidity, and/or access to medical care, and system attribution.

## RESULTS

### Anticipatory ACP Visits

**Figure 2:**
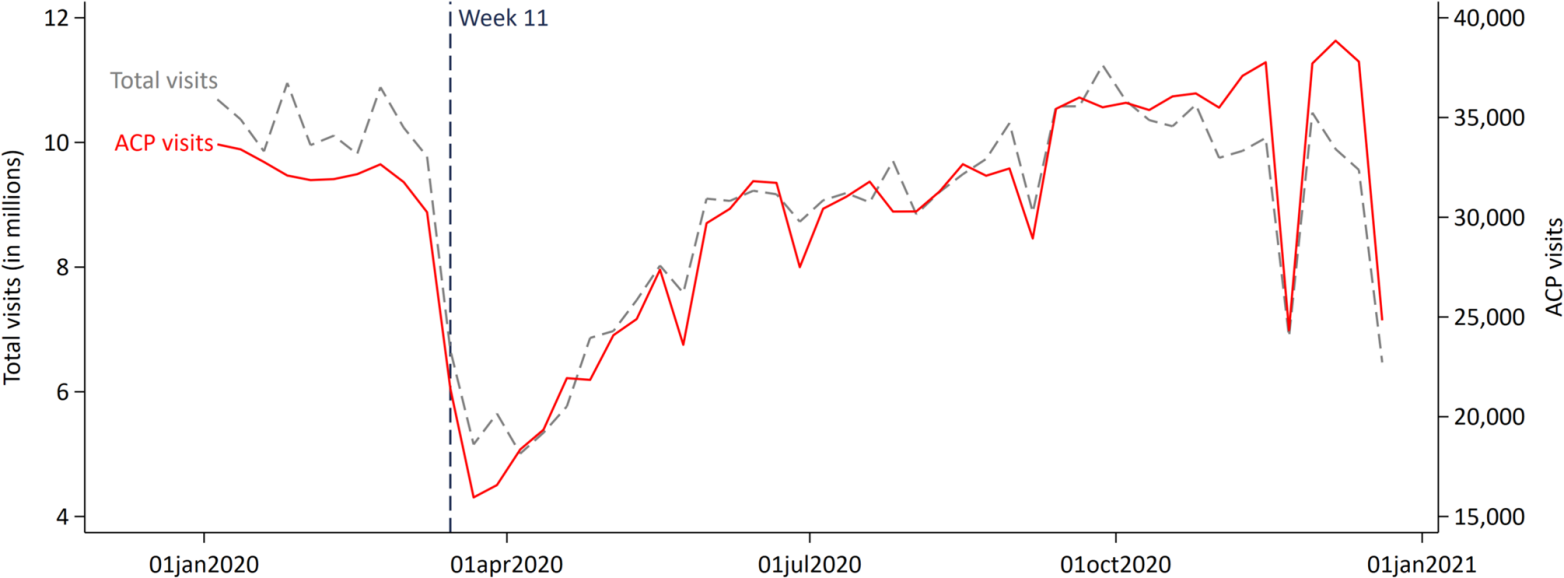
Billed encounters in 2020, by week. The dotted gray line represents all billed encounters (see left y-axis label). The red line represents the subset of those encounters that were advance care planning (ACP) encounters (see right y-axis label). The vertical dotted line represents March 15, 2020, the beginning of the COVID-19 lockdown period in the USA.

**Figure 3:**
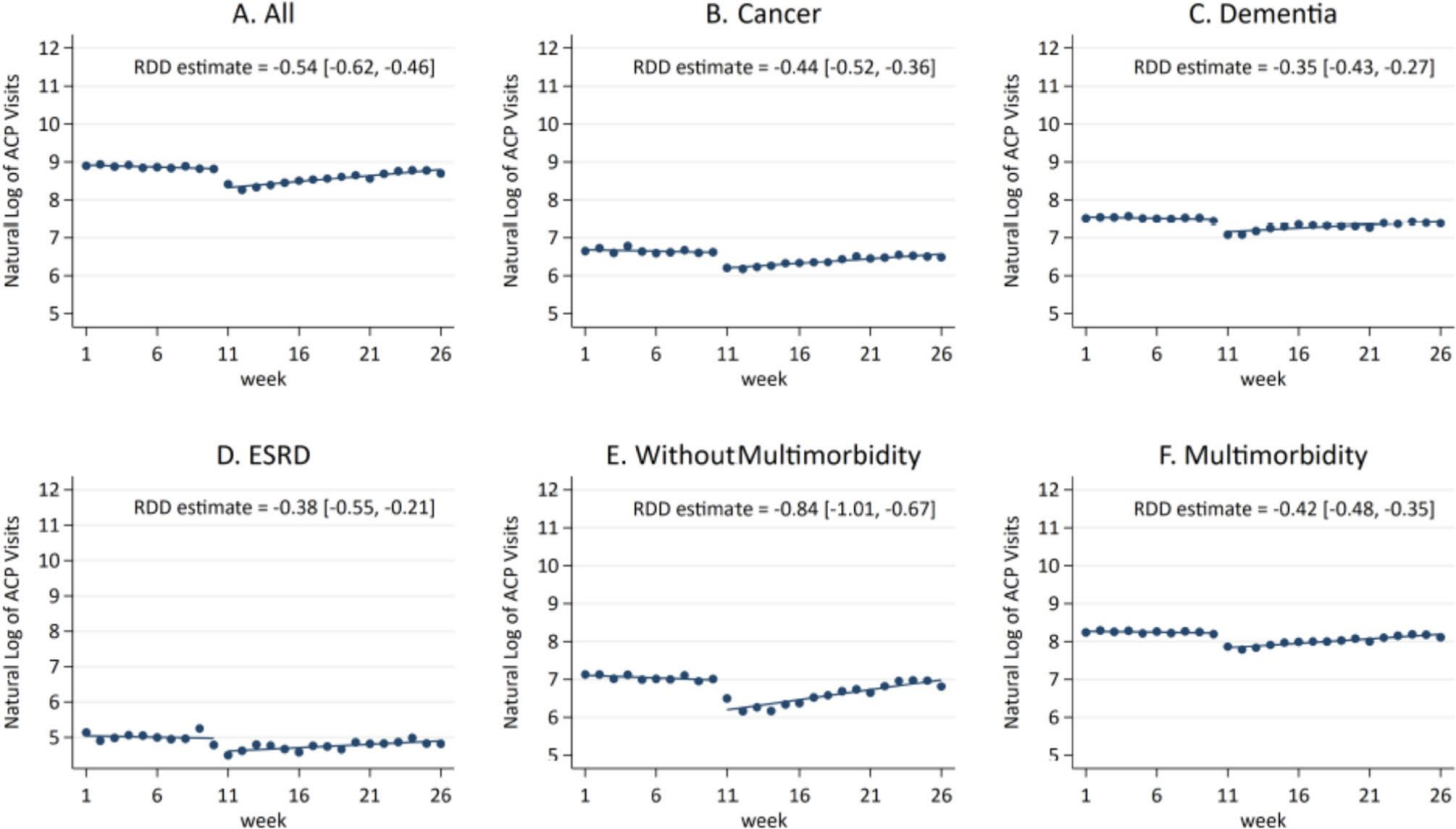
Weekly anticipatory advance care planning (ACP) visit rate, by COVID-19 mortality risk strata. (**A**) includes all beneficiaries. (**B**), (**C**), and (**D**) include subgroups of beneficiaries with poor-prognosis cancer (**B**), dementia (**C**), end-stage renal disease (ESRD) (**D**). (**E**) includes lower-risk patients (those younger than 80 with no serious chronic conditions) and (**F**) includes high-risk patients (aged 80 and older with one or more serious chronic conditions)

The full study cohort included 24,935,234 Medicare FFS beneficiaries aged 66 and older enrolled on January 1, 2020; 3,987,573 (16%) were age 80+ and had at least one serious chronic condition and 10,951,493 (44%) were < 80 with no serious chronic conditions (Table 1). There were 1,578,331 billed ACP visits during 2020, including 741,614 (47%) conducted during an AWV; 318,813 (20%) during non-AWV ambulatory visits (corresponding to our definition of “anticipatory” ACP); 302,797 (19%) during an emergency department visit or inpatient hospitalization; and 215,107 (14%) in a nursing facility. Before March 15, 2020, weekly anticipatory ACP encounters (expressed per 100,000 beneficiaries) averaged 100.5 for the whole cohort, 163.4 for those 80+ with at least one serious chronic condition, and 43.7 for those <80 with no serious chronic conditions. With the pandemic lockdown, there was an abrupt decrease in all encounters, including ACP encounters, with a steady recovery thereafter (Fig. 2). About one-quarter of the anticipatory ACP visits in the lockdown period were telehealth-delivered (Appendix). Overall, anticipatory ACP decreased by 54% (95% CI= −0.62, −0.46); the relative decline in anticipatory ACP was smaller for the highest COVID-19 mortality risk groups: multimorbidity (−42%; 95% CI= −0.48, −0.35), the presence of advanced cancer (−44%; 95% CI=−0.52, −0.36), dementia (−35%; 95% CI=−0.43, −0.27), or ESRD (−38%; 95% CI= −0.55, −0.21) (Fig. 3).

**Table 1:** Cohort Characteristics of Medicare Fee-for-Service Beneficiaries Overall, and Those Diagnosed with COVID-19, 2020

	All beneficiaries (24,935,234)		Beneficiaries with COVID-19 (1,314,986)	
	*N*	%	*N*	%
Age, years				
66–69	6,321,367	25.35	260,238	19.79
70–79	11,903,801	47.74	553,385	42.08
80+	6,710,066	26.91	501,363	38.13
Female	13,933,343	55.88	755,486	57.45
RTI race code				
American Indian/Alaskan Native	117,282	0.47	10,198	0.78
Asian Pacific Islander	708,908	2.84	28,469	2.16
Black or African American	1,652,118	6.63	116,192	8.84
Non-Hispanic White	20,531,785	82.34	1,041,546	79.21
Other	208,863	0.84	9,057	0.69
Unknown	499,730	2.00	17,961	1.37
Hispanic (any race)	1,216,548	4.88	91,563	6.96
Dual eligible (Medicare + Medicaid)	2,870,475	11.51	404,090	30.73
Comorbid conditions (weeks 0–10)				
Coronary artery disease (CAD)	563,204	2.26	46,210	3.51
Stroke	460,956	1.85	64,318	4.89
Congestive heart failure (CHF)	1,644,482	6.60	171,058	13.01
Connective tissue disorder	817,980	3.28	52,990	4.03
Chronic obstructive pulmonary disease (COPD)	1,551,492	6.22	149,736	11.39
Diabetes	2,486,214	9.97	221,812	16.87
Hematologic disorder	64,038	0.26	5,006	0.38
HIV/AIDS	25,963	0.10	2,413	0.18
Immune disorder	191,013	0.77	13,360	1.02
Liver disease	152,586	0.61	11,909	0.91
Parkinson’s or Huntington’s disease	275,379	1.10	35,269	2.68
Paralysis	81,843	0.33	9,807	0.75
Renal disease	810,350	3.25	92,364	7.02
Serious mental illness	1,155,086	4.63	163,506	12.43
Peripheral vascular disease	1,897,160	7.61	213,697	16.25
Dementia	2,084,026	8.36	396,544	30.16
Poor prognosis cancer	1,102,473	4.42	68,689	5.22
End-stage renal disease (ESRD)	180,157	0.72	27,460	2.09
HCC score*, mean (SD)	0.94 (1.52)		1.67 (2.02)	

*RTI*, Research Triangle Institute; *HIV*, human immunodeficiency virus; *AIDS*, acquired immune deficiency syndrome; *HCC*, Hierarchical condition category score

^*^Calculated based on week 0 to 10 diagnoses

### Sensitivity Analyses

These findings were qualitatively unchanged, although somewhat blunted, in sensitivity analyses including ACP delivered in nursing homes (Appendix Figure 3a) or during AWV (Appendix Figure 3b) in our definition of potentially COVID-19 anticipatory. This is because ACP visits in nursing homes were less severely impacted by the lockdown (Appendix Figure 3c) and because ACP visits during AWV were universally reduced (Appendix Figure 3d).

### Predictors of Anticipatory ACP

Being older, having two or more serious chronic conditions, or having dementia, cancer, or end-stage renal disease all strongly predicted anticipatory ACP (Table 2). These associations persisted in the supplemental model that included measures of structural disadvantage (membership in a marginalized racial group, dual Medicare-Medicaid enrollment, residential area disadvantage or rural location) and attribution to an integrated health care delivery system. Compared with non-Hispanic white persons, those who identify as Black/African American and Hispanic, and those dually enrolled in Medicaid, were more likely to receive anticipatory ACP, whereas patients living in an area with higher area deprivation, patients living in a rural region, and patients attributed to a health care system were less likely to receive anticipatory ACP (Table 2). Results were not sensitive to attribution based on the full year when compared to attribution based on weeks 0 to 10 only (data not shown).

**Table 2:** Predictors of Anticipatory ACP During the US COVID-19 Lockdown Period, Weeks 11–26, 2020

	Model 1*		Model 2^†^	
	OR	95% CI	OR	95% CI
Age (comparator: 66–70), years				
71–75	1.14	1.120–1.154	1.14	1.125–1.159
76–80	1.33	1.312–1.353	1.35	1.330–1.371
81–85	1.55	1.524–1.573	1.57	1.544–1.594
86+	1.83	1.797–1.853	1.85	1.820–1.878
Clinical risk factors				
HCC score (per 1-point increase above 0)	1.08	1.079–1.084	1.09	1.084–1.089
Dementia	2.27	2.244–2.298	2.21	2.182–2.237
Cancer	1.97	1.939–2.000	2.02	1.992–2.054
End-stage renal disease	1.57	1.513–1.623	1.43	1.384–1.486
Any ACP billing, weeks 0–10	3.63	3.782–3.920	3.48	3.634–3.766
Race (comparator: Non-Hispanic white)				
Black/African American			1.23	1.211–1.255
Hispanic ethnicity			1.25	1.225–1.278
“Other” race category			1.36	1.336–1.388
Dual Medicaid-Medicare enrollment			1.04	1.026–1.054
ADI category (referent = least disadvantaged)				
Category 2, middle			0.81	0.799–0.823
Category 3, most disadvantaged			0.87	0.857–0.892
Rural region of residence (RUCA = 10)			0.59	0.571–0.608
Integrated system-attributed (vs. independent practice)			0.73	0.718–0.732

All results are significant at the <0.0001 level

^*^Adjusted for age and comorbidities (HCC score, dementia, poor-prognosis cancer, end-stage renal disease) and prior advance care planning billing in weeks 1 to 10

^†^Adjusted for patient-level covariates in model 1 plus individual social/economic disadvantage (Medicaid insured, marginalized racial or ethnic group), health system attribution (integrated system compared to independent practice), and regional factors (area disadvantage index, rural region)

### Influence of Anticipatory ACP on Hospitalization and Mechanical Ventilation

In analyses restricted to the 1,314,986 persons diagnosed with COVID-19 during the study period, those who had received anticipatory ACP were more likely to be hospitalized in the 14 days after a billed encounter for COVID-19, but less likely to receive invasive mechanical ventilation if they were hospitalized (Table 3). Adjustment for measures of structural disadvantage and health system attribution did not change these results.

**Table 3:** Association Between Anticipatory ACP and Hospitalization, Conditional on COVID-19 Diagnosis, and Invasive Mechanical Ventilation, Conditional on COVID-19 Hospitalization

Outcome	Model 1^‡^		Model 2^§^	
	OR	95% CI	OR	95% CI
Hospitalized | COVID-19 (14d*)	1.213	1.173–1.255	1.200	1.160–1.242
Invasive mechanical ventilation | hospitalized for COVID-19 (14d*)	0.859	0.765–0.964	0.845	0.752–0.949

All results are significant at the <0.01 level

^*^Within 14 days of a billed encounter for COVID-19

^‡^Adjusted for age and comorbidities (HCC score, dementia, poor-prognosis cancer, end-stage renal disease) and prior advance care planning billing in weeks 1 to 10

^§^Adjusted for patient-level covariates in model 1 plus individual social/economic disadvantage (Medicaid insured, marginalized racial or ethnic group), the patient receives ambulatory care from an integrated health system (compared to independent practice or medical group), and regional factors (area disadvantage index, rural region)

## DISCUSSION

Billed advance care planning (ACP) visits made up a small fraction of clinician encounters in 2020. Like all encounters, billed ACP visits dropped off precipitously at the start of the pandemic lockdown in March 2020. However, the drop-off was blunted for patients at high COVID-19 mortality risk. Patients more likely to receive anticipatory ACP included those at an advanced age, with one or more serious life-limiting illnesses, from historically marginalized racial or ethnic groups, living in more advantaged ZIP codes and urban areas, and being primarily cared for by an independent practice (versus a large, integrated health system). Finally, the receipt of anticipatory ACP during lockdown was positively associated with hospitalization and negatively associated with invasive mechanical ventilation after a COVID-19 diagnosis post-lockdown.

These findings suggest providers were using COVID-19 risk-based targeting during lockdown. In early 2020, concerns regarding fatality rates for certain sub-groups likely led clinicians to prioritize their outreach to patients for whom treatment burden was thought to outweigh benefit. The association of anticipatory ACP with hospitalization with COVID-19 suggests clinical confirmation of risk targeting — those who received anticipatory ACP were more likely to be hospitalized when they had COVID-19. Medicare beneficiaries prefer to avoid mechanical ventilation at the end of life.^13^ Given that respiratory failure due to COVID in the early days of the pandemic was considered likely terminal, the reduction in invasive mechanical ventilation associated with anticipatory ACP suggests that the ACP was indeed effective at reducing unwanted life-supporting treatment. This finding is important, given the mixed evidence regarding the influence of ACP on distal outcomes such as end-of-life treatment receipt,^14^ and the ongoing controversy in the field regarding the value of ACP.^15^ Additionally, it underscores the need for effective identification and dissemination of clinical risk factors in the next pandemic to guide anticipatory decision making.

The correlation of structural factors with anticipatory ACP offers a more nuanced picture. Persons from historically marginalized groups that were known to be dying at higher rates^16^ appear also to have been targeted for anticipatory ACP. This may have been a result of direct targeting based on predicted clinical risk within a physician’s panel. Alternatively, it could have been attributable to the concentration of such patients in the panels of practitioners who heeded the call to action, as has been demonstrated through other population-health promoting practices by clinicians in minority-serving practices.^17,18^ Using a different dataset, we found that ACP billing during an inpatient stay with COVID-19 (representing reactive, not anticipatory, ACP) was similar across persons from different racial and ethnic backgrounds.^19^ Our finding is interesting, given the well-documented observation that persons from minoritized backgrounds are, in general, less likely to have completed ACP.^20^ The correlation of anticipatory ACP with geography (urban, advantaged areas) and health care organization (attribution to an independent medical practice or group) may offer some insight into the types of physicians who were drawn to this work in the early weeks of the pandemic. Alternative speculation for why patients attributed to large systems had lower rates of anticipatory ACP could be that they were less likely to bill than those in smaller, more resource-strapped groups or that they were called to prepare for waves of seriously ill patients and had less availability for the work compared with smaller practices at which non-essential visits were curbed or ended.

While there were many professional calls for targeted ACP during the COVID-19 pandemic, only a handful of studies to date have explored the effects of the COVID-19 pandemic on ACP^21–23^ or the effect of ACP on treatment. A single-center study of patient portal users found increased use of the ACP portal tool in the early phase of the pandemic.^16^ One retrospective cohort analysis of 21,962 records from an electronic palliative care coordination database found that documented elements of ACP were associated with dying in the preferred location during the pandemic.^24^ One integrated delivery system that implemented team-based ACP during the pandemic demonstrated that anticipatory ACP was associated with reductions in ICU use and costs, conditional on ICU admission.^25^ We anticipate that additional studies will emerge seeking to explore the complex effects of the COVID-19 pandemic on ACP and outcomes, particularly as prognostic understanding, prevention, and treatment options evolved over the pandemic.

This study has many strengths, including a national sample, robust analytic design, and novelty. It also has many weaknesses. Chiefly, the sensitivity of ACP billing for ACP conversations is likely very low,^26^ and even more so during the pandemic. Anecdotally, we heard from physicians making these outreach phone calls in the early weeks of the pandemic that they did not reliably bill for their ACP visits. Additionally, it is not possible to affirm whether a billed ACP visit is truly anticipatory. Many of the ACP visits we excluded in our base case analysis could have been COVID-19 anticipatory, especially those conducted during annual wellness visits or in the nursing home setting. We were sufficiently concerned that billed ACP in nursing facility settings might be a mix of anticipatory and reactive conversations that we elected to exclude those conducted in the nursing home setting in our base case. However, we conducted sensitivity analysis in recognition of uncertainty, which suggests including these additional ACP visits would not have changed our primary results. Also related to billing and coding, COVID-19 diagnosis is also not likely a sensitive measure of having the disease, and mechanical ventilation codes are specific but not sensitive for mechanical ventilation.^27^ If the misspecification was not systematic, it would tend to bias our findings toward the null. For us to have observed the results we did due to systematic misspecification, providers would have been more likely to have billed ACP for higher- versus lower-risk COVID-19 patients, and lower-risk patients (or those who did not want life support in the event of hospitalization) would have had to have been more likely to present for medical care and COVID-19 testing when ill. There are recognized weaknesses with population-based studies using Medicare fee-for-service, including lack of generalizability to other populations (including those covered by Medicare Advantage and persons without Medicare coverage), lack of detail for classifying clinical risk, and other forms of misspecification from using patient ZIP code of residence (e.g., inference of social risk). Finally, we do not know whether these ACP discussions resulted in more goal-concordant care.

## CONCLUSIONS

Faced with the crisis of the pandemic, providers appeared to follow guidelines to target ACP to high-risk patients, which seemed to impact the intensity of treatment received by these high-risk patients in the event of a COVID-related hospitalization. The implications for future clinical practice, as with the myriad stories of innovation and initiative during the pandemic, are that providers can self-organize to benefit the patients they serve when they are “unleashed” from system-based constraints. Furthermore, it highlights the critical role that effective identification and dissemination of clinical risk factors have during public health emergencies to guide anticipatory decision making.

## Supplementary Information

Below is the link to the electronic supplementary material.

Supplementary file1 (DOCX 1243 KB)

Supplementary file2 (XLSX 136 KB)

## Data Availability

Raw counts of ACP visits drawn from Centers for Medicare and Medicaid Services (CMS) claims data are available in a supplementary appendix. The raw CMS claims data used to create these counts and to conduct the analyses herein are available to researchers via CMS' Virtual Research Data Center (VRDC; see https://resdac.org/). Our analytic code will be made available at https://dataverse.dartmouth.edu/.
