# Supplementary material for: Anticipatory Advance Care Planning Visits and COVID-19 Treatment Intensity Among Medicare Fee-for-Service Beneficiaries: A Retrospective Observational Study
Source: J Gen Intern Med. 2025 Dec 12;41(8):2098–107. doi: 10.1007/s11606-025-09638-9 (PMC12890009; doi:10.1007/s11606-025-09638-9)

APPENDIX FIGURES

Appendix Figure 2 – Weekly ACP visits in 2020, by type


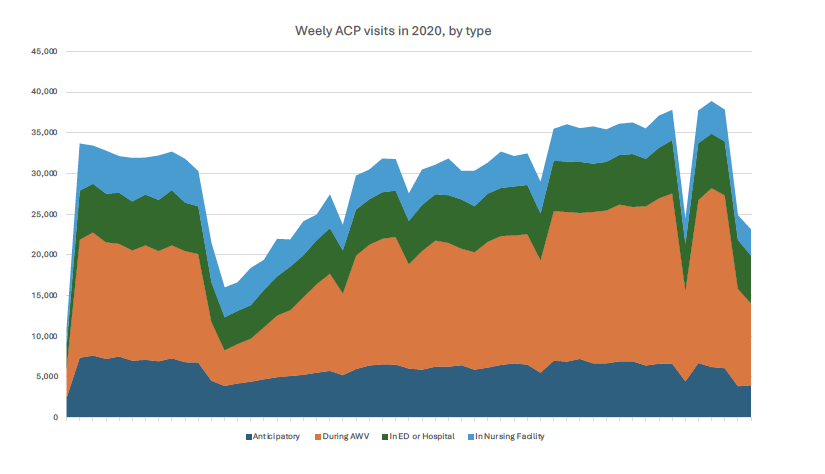


Appendix Figure 3a – Sensitivity analysis: Adding nursing facility ACP visits to anticipatory ACP visits


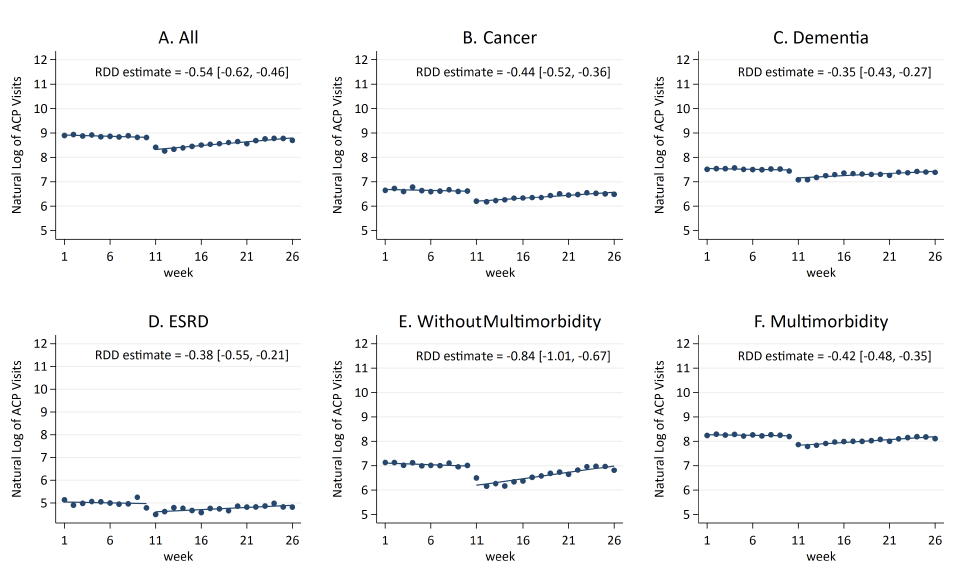


Appendix Figure 3b – Sensitivity analysis: Nursing home visits only


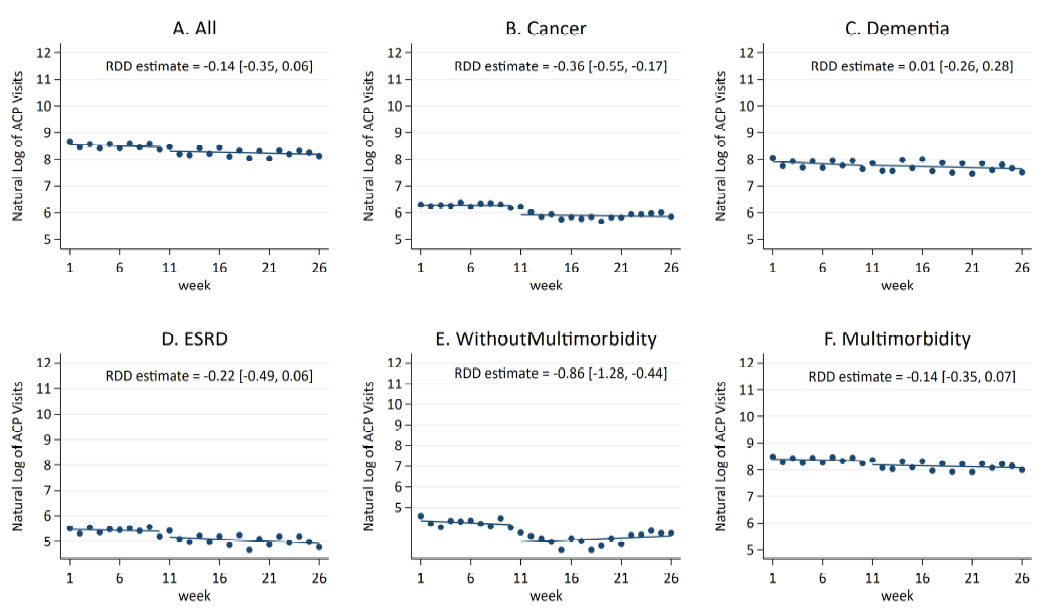


Appendix Figure 3c – Sensitivity analysis: Adding AWV ACP visits to anticipatory ACP visits


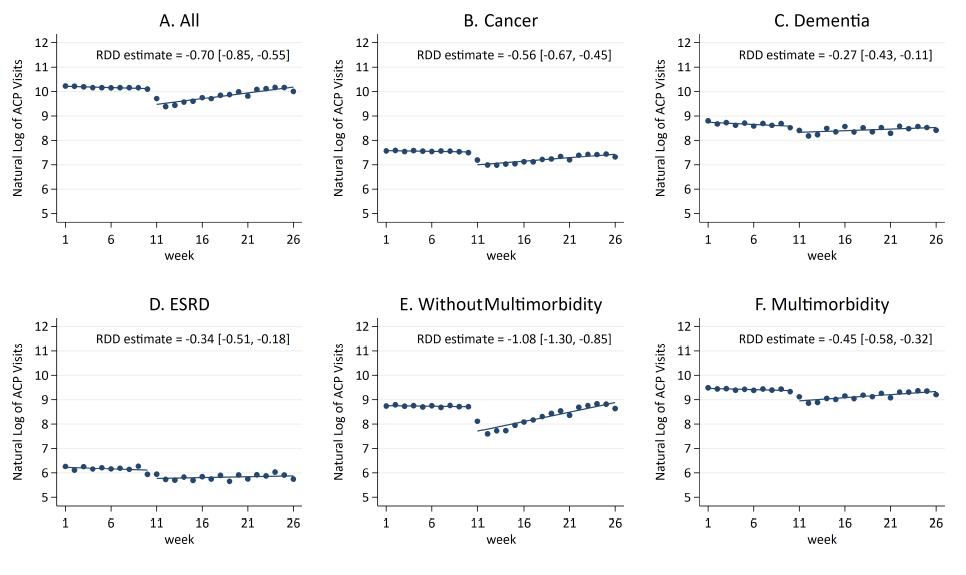


Appendix Figure 3d – Sensitivity analysis: ACP during AWV only


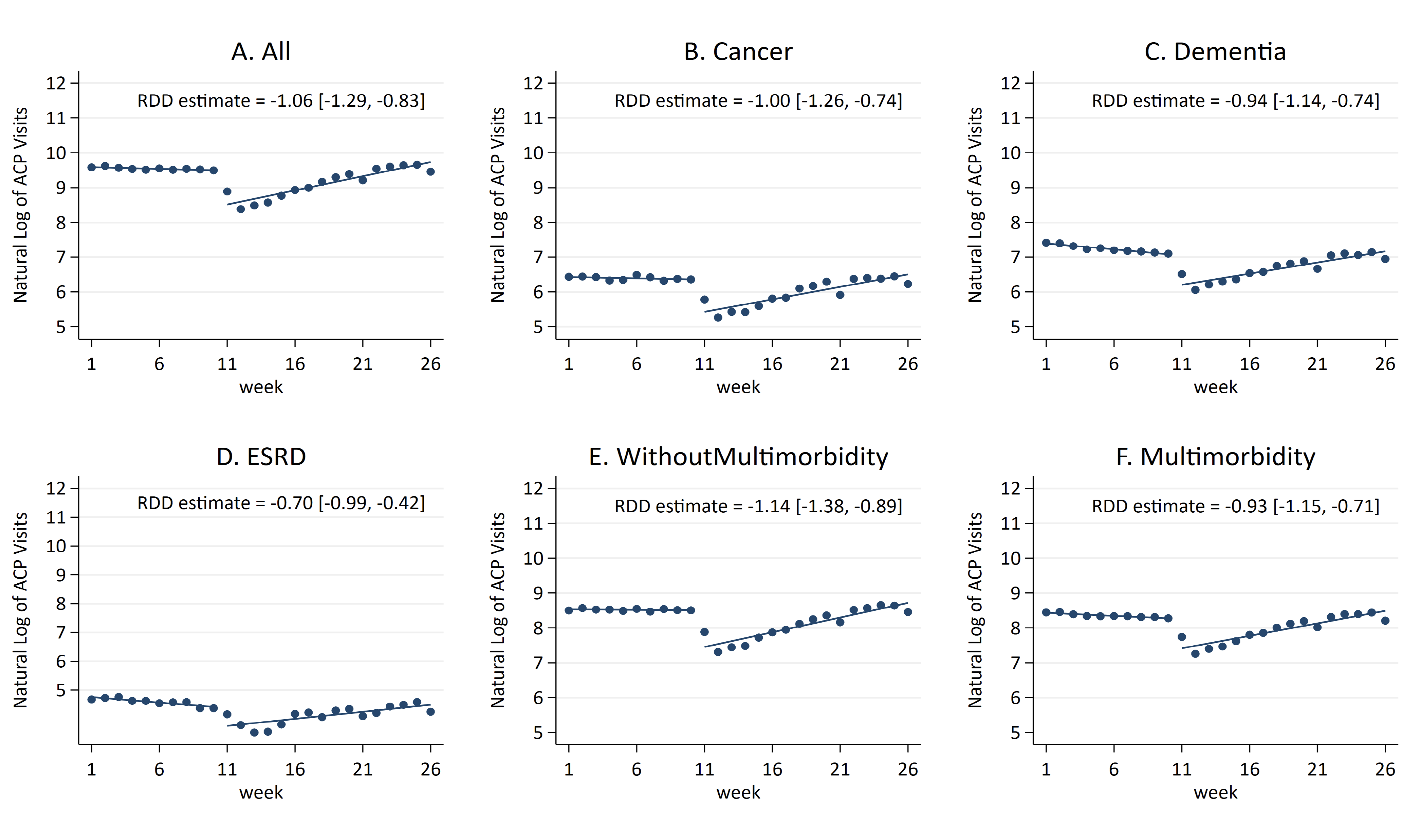

Supplement: Supplementary file 1 — Supplementary file1 (DOCX 1243 KB) [file 11606_2025_9638_MOESM1_ESM.docx]
